# Supplementary material for: Thermodynamic and structural investigation of oily wastewater treatment using peach kernel and walnut shell based activated carbon
Source: PLoS One. 2024 May 15;19(5):e0297024. doi: 10.1371/journal.pone.0297024 (PMC11095765; doi:10.1371/journal.pone.0297024)
Supplement: Attachment — Submitted filename: ResponseToReviewers.docx [file pone.0297024.s001.docx]

**Response to the Reviewers**

The authors would like to thank the editor and the reviewers for their thoughtful comments and efforts toward improving our manuscript. A point-by-point response to the comments of the reviewers is as follows.

**Reviewer 1**

**Comment 1:** Abstract required further refinement to ensure better understandings (i.e Line 28-30, line 38-40) and contained grammatical errors. Need to proofread the manuscript before submitting.

**Answer:**

Thank you very much for your comment. The comment was applied and the Abstract section was further refined to ensure better understandings which is highlighted (Line 26-29 and Line 36-40). Furthermore, the manuscript was checked grammatically and was edited properly. The added text is as follow:

“This present study found that the peach activated carbon with a chemical process had the highest hardness (97%) compared to other types of activated carbon and the physical activated walnut carbon active has obtained the lowest hardness value (87%).”

“Furthermore, the study investigated the impact of temperature and pH on the adsorption of various synthesized oily wastewater. The findings revealed that an increase in temperature led to a decrease in adsorption capacity, while the adsorption rate increased with increasing temperature. Moreover, it was found that a decrease in adsorption observed when the adsorbent is negatively charged and the pH is lowered”

**Comment 2:** Introduction part need to be fully revised especially the continuity in sentences and between paragraphs. Some of the statements are too broad.

**Answer:**

Thank you very much for your comment. The comment was applied and the Introduction part was revised and rewritten to ensure better understandings which is highlighted in the manuscript. Please see Introduction section and the highlighted text.

**Comment 3:** Justify the statement on chemical activation process (Line 93-96). There are processes that involve two-step synthesis as well. Some chemical activation uses cost-effective activated carbon production; hence the statement might be invalid. Please justify further.

**Answer:**

Thank you for your thoughtfulness. Yes, you are right, some chemical activation uses cost-effective activated carbon production. But in general, it can be said that the chemical method uses more expensive materials; although there are some exceptions. The statement was edited as follows (Line 91-93):

“Generally, the chemical method uses more expensive materials and on a smaller scale than the physical method, however, some chemical activation uses cost-effective activated carbon production”

**Comment 4:** If there is no-in depth research on said topic, what did most people research and review on? Strengthen the novelty.

**Answer:**

Thank you very much for your comment. It is important to note that the lack of in-depth research on a topic does not necessarily mean that there has been no research carried out on an area of study. It may indicate the need for further research and exploration in this area. In other words, this sentence means that the results of the works of other researchers should be placed next to each other and by covering the research gaps, a new and original result should emerge in order to open new horizons of science. Exactly what this work has tried to do to understand the causes of the difference in surface adsorption characteristics of the obtained carbon active with different precursors and different activation processes. However, in order to avoid misunderstanding, it was changed as below:

“Despite the numerous articles on activated carbon with different precursors in the adsorption process, more extensive research is needed to understand the causes of differences in the surface adsorption characteristics of activated carbon with different precursors and different activation processes.”

**Comment 5:** “FTIR and XRD analyzes and SEM images have been used to identify the functional groups of the synthesized activated carbon surface, and to measure the pore structure of the synthesized carbon active” Which characterization is for which, revise the sentence for better clarity.

**Answer:**

Thank you very much for your comment. The comment was applied and the sentence was revised as follows for better clarity (Line 100-103) :

“FTIR and XRD analyzes have been used to identify the functional groups of the synthesized activated carbon surface, and the SEM images were applied to measure the pore structure of the synthesized carbon active”

**Comment 6:** In section 2.2.2, author mentioned on chemical activating agent of phosphoric acid and ZnCl2, but at the end didn’t conclude which one is being used in the methodology part. Amend the section accordingly.

**Answer:**

Thank you very much for your comment. In this work ZnCl_2_ was applied as the activating agent. In order to clarify, the comment was applied and the manuscript was edited as follows (Line 153-157):

“To produce activated carbon, chemical activating agent of phosphoric acid and ZnCl2 can be used; in order to use a chemical process with phosphoric acid as a chemical activator, carbon material can be mixed with phosphoric acid, and then this mixture is put into a rotary kiln, after the carbonization, the activated carbon can be reached at ambient temperature. However, in this work ZnCl2 was applied as the activating agent.”

**Comment 7:** The section related to synthesis need to be revised with the addition of comparison of previous work in table form.

**Answer:**

Thank you very much for your comment. The comment was applied and a table was added to the manuscript to compare the previous works results with this work (Table 2). The most important functional parameters of the synthesized adsorbents of the previous works, i.e., the specific surface area and the iodine number, are compared in Table 2.

**Table (2)** The comparison of Specific Surface and Iodine Number of activated carbon of the previous works

| **Adsorbent** | **Specific Surface (m^2^/gr)** | **Activation Method** | **Iodine Number** | **Ref** |
| --- | --- | --- | --- | --- |
| Cellulose Wastes | 1100 | Chemical | 1080 | (43) |
| Almond | 900 | Chemical | 850 | (44) |
| Walnut | 840 | Chemical | 900 | (44) |
| natural zeolite | 198 | Chemical | 190 | (45) |
| Oil sludge | 120 | Chemical | 401 | (46) |

**Comment 8:** Should provide surface morphology images (SEM) to strengthen the activated carbon properties rather than just put in table.

**Answer:**

Thank you very much for your comment. In this manuscript, an attempt has been made to conduct a comprehensive study on the synthesis of active carbon; for this reason, many experiments and analyzes have been performed and many results have been obtained, which were presented in the most possible compact way. However, in this manuscript, there are 12 figures and 8 tables; therefore, in order to reduce the number of tables and figures, SEM images have not been presented and only the results of the SEM analysis have been presented in one table along with other activated carbon properties.

**Comment 9:** More references need especially comparing the result in discussion.

**Answer:**

Thank you very much for your comment. The comment was applied and more references (REF 43-48) were used in the results and discussion section to strengthen the results.

**Reviewer 2**

**Comment 1:** BET data should be supplemented.

**Answer:**

Thank you very much for your comment. The specific surface area as a characteristic of synthesized activated carbons was presented in Table 1 which is determined using BET analysis. The manuscript lacked information on the method used to investigate the specific surface area. To address this, it was added that the BET analysis was applied to determine the specific surface area of the synthesized carbon actives. (Line 101-102)

**Comment 2:** The authors have performed the measurement at different temperatures. The authors may need to perform the required thermodynamic analysis, since the title has been stated so.

**Answer:**

Thank you very much for your comment. As you mentioned, the manuscript aims to investigate the thermodynamics of oily wastewater treatment using activated carbon. To achieve this goal, isotherm models such as Langmuir, Freundlich, and Temkin have been used. Several studies have been conducted to understand the adsorption behavior of activated carbon using these models. The thermodynamic analysis of carbon active using these isotherm models provides a comprehensive understanding of the adsorption mechanism and helps in optimizing the adsorbent amount in a process. In other words, when the adsorbent is placed in contact with an oily solution, the oil concentration on the adsorbent surface increases until reaching thermodynamic equilibrium and then stabilizes at an equilibrium state. To understand the adsorption mechanism thermodynamically and to optimize the required adsorbent amount in a process, the isotherm models was applied. The obtained equilibrium value, known as the adsorption isotherm, is the primary basis for the design of adsorption systems. Adsorption isotherms are a powerful tool for the thermodynamic analysis of activated carbon; because the adsorption isotherms can be used to determine the adsorption capacity of activated carbon and to analyze the thermodynamics of the adsorption process.

**Comment 3:** How does "no-in depth" research on the cause of the difference in surface adsorption characteristics help in this study?

**Answer:**

Thank you very much for your comment. It is important to note that the lack of in-depth research on a topic does not necessarily mean that there has been no research carried out on an area of study. It may indicate the need for further research and exploration in this area. In other words, this sentence means that the results of the works of other researchers should be placed next to each other and by covering the research gaps, a new and original result should emerge in order to open new horizons of science. Exactly what this work has tried to do to understand the causes of the difference in surface adsorption characteristics of the obtained carbon active with different precursors and different activation processes. However, in order to avoid misunderstanding, it was changed as below:

“Despite the numerous articles on activated carbon with different precursors in the adsorption process, more extensive research is needed to understand the causes of differences in the surface adsorption characteristics of activated carbon with different precursors and different activation processes.”

**Comment 4:** Based on "3", will the analysis be different if the method of activation is changed?

**Answer:**

Thank you very much for your comment. In the syntheses of all types of adsorbents, the main goal is to produce adsorbents with a larger specific surface area and, as a result, greater potential for adsorption which can be studied using BET analysis and the iodine number determination, respectively. The most important functional parameters of the adsorbents of the previous works, *i.e.*, the specific surface area and the iodine number, are compared in Table 2.

**Comment 5:** Formatting issue in Table 2.

**Answer:**

Thank you very much for your comment. The comment was applied and the Table 2 (which is changed to Table 3 in the new manuscript) was edited properly.

**Comment 6:** How do the performance of the studied adsorbents reported as compared to the literature studies?

**Answer:**

Thank you very much for your comment. The comment was applied and a table was added to the manuscript to compare the previous works results with this work (Table 2). The most important functional parameters of the adsorbents of the previous works, *i.e.,* the specific surface area and the iodine number, are compared in Table 2.
