# Supplementary material for: Thermodynamic and structural investigation of oily wastewater treatment using peach kernel and walnut shell based activated carbon
Source: PLoS One. 2024 May 15;19(5):e0297024. doi: 10.1371/journal.pone.0297024 (PMC11095765; doi:10.1371/journal.pone.0297024)
Supplement: Attachment — Submitted filename: Authors Response to Reviewers.docx [file pone.0297024.s002.docx]

1. We notice that your manuscript file was uploaded on February 18, 2023. Please can you upload the latest version of your revised manuscript as the main article file, ensuring that does not contain any tracked changes or highlighting.  This will be used in the production process if your manuscript is accepted. Please follow this link for more information: <http://blogs.plos.org/everyone/2011/05/10/how-to-submit-your-revised-manuscript/>

**Reply:** We uploaded the last version of manuscript without any tracked changes or highlighted text.

2. Thank you for updating your data availability statement. You note that your data are available within the Supporting Information files, but no such files have been included with your submission. At this time we ask that you please upload your minimal data set as a Supporting Information file, or to a public repository such as Figshare or Dryad. Please also ensure that when you upload your file you include separate captions for your supplementary files at the end of your manuscript.

As soon as you confirm the location of the data underlying your findings, we will be able to proceed with the review of your submission.

**Reply:** We understand the importance of data accessibility and transparency in research. We would like to assure you that the data supporting the findings of our study are presented through the figures and tables included in the manuscript. These figures and tables contain the necessary information to reproduce the study results. However, we acknowledge that providing the data in a more accessible format would be beneficial for readers. To address this concern, we propose the providing the data upon reader request option for data availability. In this way, we are willing to share the data with interested readers upon request. We will ensure that the data are made available in a timely manner and provide the necessary contact information for readers to request access. We added below section to the paper:

**Data Availability Statement:** The data presented in this study are available on request from the corresponding author.

3. We note that the grant information you provided in the 'Funding Information' and 'Financial Disclosure' sections do not match.

When you resubmit, please ensure that you provide the correct grant numbers for the awards you received for your study in the 'Funding Information' section.

Please include your amended statements within your cover letter; we will change the online submission form on your behalf.

**Reply:** The exact information of funding and acknowledgment are as follow:

**Acknowledgments:**

The authors acknowledge the anonymous reviewers for their valuable suggestions that helped improve the quality of the manuscript.

**Funding:**

This work was funded by the King Khalid University, Abha, Saudi Arabia. The appreciation to the Deanship of Scientific Research at King Khalid University for funding this work through Large Groups Project under grant number (R.G.P. 2/57/44).

4. Please include a figure label and title for Figure 1 to 12 in your main manuscript.

**Reply:** We added label and title for all figures.

5. Please note that funding information should not appear in the Acknowledgments section or other areas of your manuscript. We will only publish funding information present in the Funding Statement section of the online submission form. Please remove any funding-related text from the manuscript.

**Reply:** We revised these two sections as follow:

**Acknowledgments:**

The authors acknowledge the anonymous reviewers for their valuable suggestions that helped improve the quality of the manuscript.

**Funding:**

This work was funded by the King Khalid University, Abha, Saudi Arabia. The appreciation to the Deanship of Scientific Research at King Khalid University for funding this work through Large Groups Project under grant number (R.G.P. 2/57/44).

6. We note that several of your files are duplicated on your submission. Please remove any unnecessary or old files from your revision, and make sure that only those relevant to the current version of the manuscript are included.

**Reply:** We removed unnecessary or old files from our revision that were duplicated.
